# Acute molecular effects of pressure‐controlled intermittent coronary sinus occlusion in patients with advanced heart failure

**DOI:** 10.1002/ehf2.12354

**Published:** 2018-09-19

**Authors:** Werner Mohl, Ernest Spitzer, Robert M. Mader, Vilas Wagh, Filomain Nguemo, Dejan Milasinovic, Alem Jusić, Cesar Khazen, Edit Szodorai, Beatrice Birkenberg, Gert Lubec, Juergen Hescheler, Patrick W. Serruys

**Affiliations:** ^1^ Department of Cardiac Surgery Medical University of Vienna Vienna Austria; ^2^ Department of Cardiology, Thoraxcenter Erasmus University Medical Center Rotterdam The Netherlands; ^3^ Department of Medicine I Comprehensive Cancer Center of the Medical University of Vienna Vienna Austria; ^4^ Massachusetts General Hospital, Harvard Medical School Boston MA USA; ^5^ Center of Physiology and Pathophysiology, Institute of Neurophysiology University of Cologne Cologne Germany; ^6^ Department of Cardiology Clinical Center of Serbia Belgrade Serbia; ^7^ Department of Molecular Neurosciences, Center for Brain Research Medical University Vienna Vienna Austria; ^8^ Department of Anesthesiology and General Intensive Care Medical University of Vienna Vienna Austria; ^9^ Department of Pharmaceutical Chemistry Faculty of Life Sciences University of Vienna Vienna Austria; ^10^ International Centre for Circulatory Health, NHLI, Imperial College London London UK

**Keywords:** Embryonic recall, PICSO, Cardiac regeneration, Heart failure

## Abstract

**Aims:**

Cardiac repair has steered clinical attention and remains an unmet need, because available regenerative therapies lack robust mechanistic evidence. Pressure‐controlled intermittent coronary sinus occlusion (PICSO), known to induce angiogenetic and vasoactive molecules as well as to reduce regional ischemia, may activate endogenous regenerative processes in failing myocardium. We aimed to investigate the effects of PICSO in patients with advanced heart failure undergoing cardiac resynchronization therapy.

**Methods and results:**

Eight out of 32 patients were treated with PICSO, and the remainder served as controls. After electrode testing including left ventricular leads, PICSO was performed for 20 min. To test immediate molecular responses, in both patient groups, coronary venous blood samples were taken at baseline and after 20 min, the time required for the intervention. Sera were tested for microRNAs and growth factors. To test the ability of up‐regulated soluble factors on cell proliferation and expression of transcription factors [e.g. Krüppel‐like factor 4 (KLF‐4)], sera were co‐cultured with human cardiomyocytes and fibroblasts. As compared with controls, significant differential expression (differences between pre‐values and post‐values in relation to both patient cohorts) of microRNA patterns associated with cardiac development was observed with PICSO. Importantly, miR‐143 (*P* < 0.048) and miR‐145 (*P* < 0,047) increased, both targeting a network of transcription factors (including KLF‐4) that promote differentiation and repress proliferation of vascular smooth muscle cells. Additionally, an increase of miR‐19b (*P* < 0.019) known to alleviate endothelial cell apoptosis was found, whereas disadvantageous miR‐320b (*P* < 0.023) suspect to impair expression of c‐myc, normally provoking cell cycle re‐entry in post‐mitotic myocytes and miR‐25 (*P* < 0.023), decreased, a target of anti‐miR application to improve contractility in the failing heart. Co‐cultured post‐PICSO sera significantly increased cellular proliferation both in fibroblasts (*P* < 0.001) and adult cardiomycytes (*P* < 0.004) sampled from a transplant recipient as compared with controls. Adult cardiomyocytes showed a seven‐fold increase of the transcription factor KLF‐4 protein when co‐cultured with treated sera as compared with controls.

**Conclusions:**

Here, we show for the first time that PICSO, a trans‐coronary sinus catheter intervention, is associated with an increase in morphogens secreted into cardiac veins, normally present during cardiac development, and a significant induction of cell proliferation. Present findings support the notion that epigenetic modifications, that is, haemodynamic stimuli on venous vascular cells, may reverse myocardial deterioration. Further investigations are needed to decipher the maze of complex interacting molecular pathways in failing myocardium and the potential role of PICSO to reinitiate developmental processes to prevent further myocardial decay eventually reaching clinical significance.

## Introduction

Chronic heart failure accounts for a substantial amount of morbidity and mortality worldwide.^1^ Although not yet an established therapy, structural myocardial regeneration may benefit an enormous population.^2^ Pressure‐controlled intermittent coronary sinus occlusion (PICSO), a trans‐coronary sinus catheter intervention, was primarily developed to salvage jeopardized myocardium during acute ischemic syndromes. It has been shown that PICSO intervention leads to the activation of important growth factors such as vascular endothelial growth factor and interleukin 6, which in turn may stimulate cardiac regeneration via cardiomyocyte proliferation.^3^ Recently, a report on PICSO in acute heart failure patients suggests recovery beyond myocardial salvage.^4^ We hypothesized that PICSO with its periodic ventricularization of the coronary venous compartment may modify the concentration of molecules relevant to commence developmental pathways.

## Aims

We aimed to investigate the effects of PICSO on the release of soluble molecules linked to myocardial development through direct comparisons among pre‐intervention and post‐intervention sera and equivalent control sera. Furthermore, we aimed to investigate the effects of released molecules on cellular events.

## Methods

### Study population

Patients with advanced heart failure undergoing cardiac resynchronization therapy (CRT) implantation and older than 18 years of age were eligible for this study. Exclusion criteria included the presence of prosthetic heart valves or indication for valve replacement, unstable angina pectoris, known bleeding disorder, chronic steroid therapy, cancer, infectious diseases of any cause, pregnancy, and haemodynamic or electrical instability. All subjects provided written informed consent. The study protocol and all procedures involved complied with the Declaration of Helsinki and were approved by the Ethics Committee of the Medical University of Vienna.

### Study protocol

The study took place in the electrophysiology operating suite during CRT device implantation. After left ventricular electrode placement within the coronary veins and electrical threshold testing, a specially designed coronary sinus catheter with a curved tip and a helium inflatable balloon was used for the application of PICSO (Contract Medical International, Dresden, Germany). Periodic coronary sinus pressure (CSP) elevation was achieved using an automated PICSO controller (ARC Seibersdorf Research GmbH, Seibersdorf, Austria).

### Analysis of the collected sera

**Figure 1:**
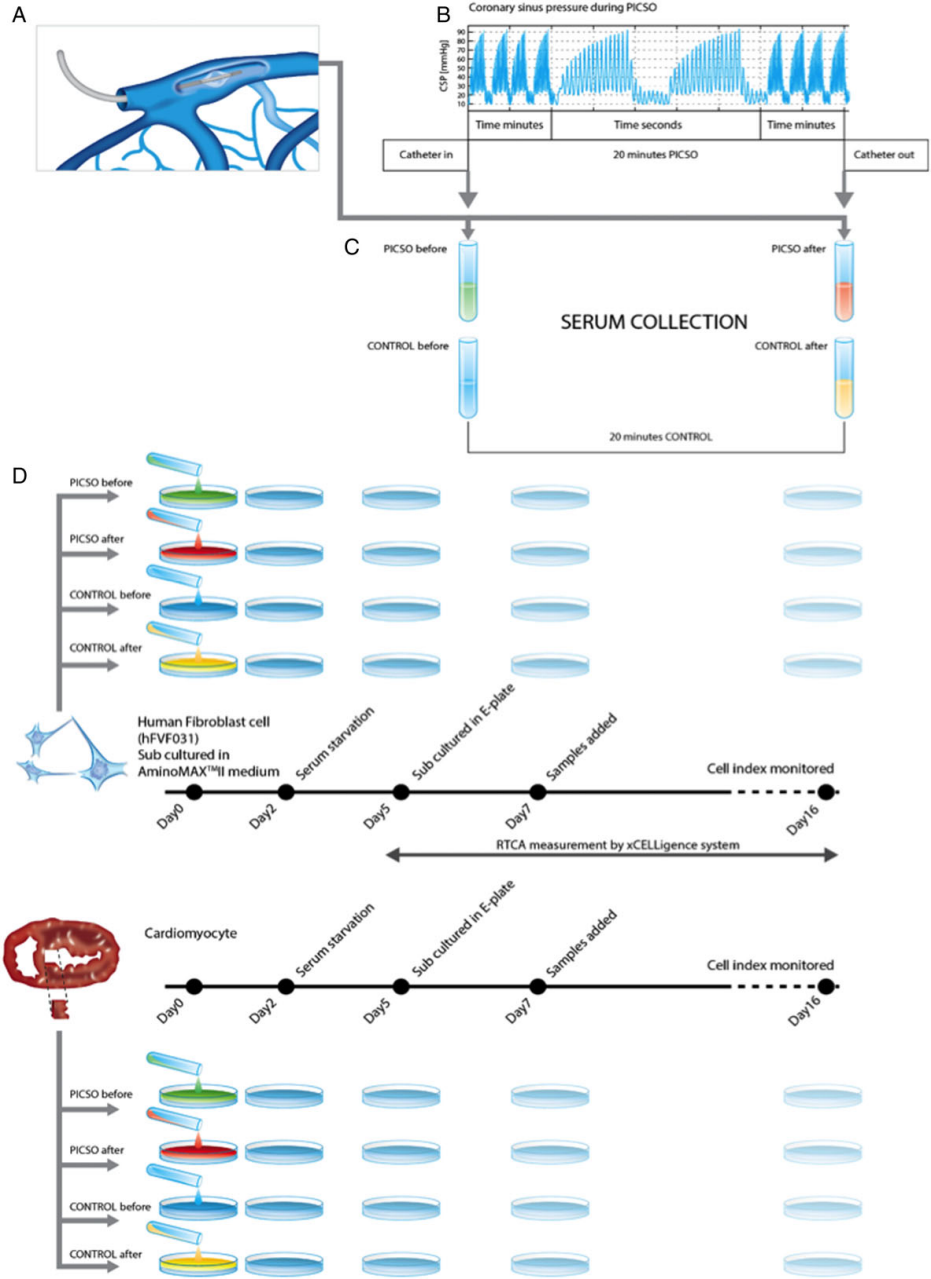
Study protocol and serum collection. (A) After advancement of a coronary sinus catheter and temporary pressure‐controlled occlusion (PICSO) of a major portion of outflow from coronary veins, pressures are increased as seen in (B); systolic pressures peaks are thought to be the driving force to activate venous endothelium. (C) Immediately frozen to −80°, (D) human fibroblasts and cardiomyocytes are co‐cultured with patient sera to estimate the proliferative power of each serum. After primary culture, cells underwent a starving period before co‐cultured with patient serum.

To investigate the effects of PICSO on the release of soluble factors, coronary sinus blood samples before and after 20 min of treatment or the respective time in control patients were taken. Samples were then centrifuged and shock frozen for storage at −80°C. MicroRNA and proteomics analyses were performed subsequently. As shown in *Figure* *1*, sera from coronary sinus blood were co‐cultured with a mixture of human fibroblasts and adult cardiomyocytes stemming from the septum of a failing heart from a patient undergoing heart transplantation and commercially available human fibroblasts.

### Statistical analysis

Statistical analyses were performed using SPSS for Windows V20 (SPSS Inc., Chicago, IL, USA). The measurements are expressed as mean ± standard deviation. Preoperative patients' characteristics were compared using χ^2^ tests; in terms of *n* < 5 in one group, the Fisher's exact test was performed. An alpha error below *P* = 0.05 was considered indicative of a statistically significant difference.

## Results

### Study population

Thirty‐two patients after successful CRT implantation were included in the study. Eight patients were investigated with PICSO for 20 min, and 24 patients constituted the control group. Between the two groups, no significant differences were noted at baseline.

## Demographics and clinical data

*Table* 1 depicts demographics and clinical data, showing comparable parameter in both groups.

**Table 1:** summary of demographics and clinical data

	PICSO (*n* = 8)	Control (*n* = 24)	*P*‐value (χ^2^ and Fisher's exact test^b^)
Demographics			
Age (years) ± SD (*n*)	73.25 ± 10.78	64.75 ± 11.0	0.076^a^
Gender (*N* and % male)	7	17	0.346
	87.5%	70.83%	
Ejection fraction (%)	23.13 ± 6.94	25.70 ± 8.20	0.433^a^
Clinical history			
Diabetes	2	6	1
	25%	25%	
COPD	1	7	0.642
	12.5%	29.17%	
Hyperlipidaemia	6	17	1
	75%	70.83%	
Hypertension	8	20	0.217
	100%	83.33%	
Smoking	3	6	0.654
	37.5%	25%	
Previous stroke	1	1	0.444
	12.5%	4.17%	
Previous myocardial infarction	3	12	0.691
	37.5%	50%	
Valvular disease	3	10	1
	37.5%	41.67%	
Chronic atrial fibrillation (anamnesis)	1	10	0.209
	12.5%	41.67%	
Atrial fibrillation at admission	0	4	0.550
	0%	16.67%	
Dilated CMP	5	9	0.217
	62.5%	37.5%	
Ischaemic CMP	3	14	0.423
	37.5%	58.33%	
Valvular CMP	0	1	1
	0%	4.17%	
Conduction abnormalities			
Left bundle branch block	5	17	0.66
	62.5%	70.83%	
Right bundle branch block	0	3	0.555
	0%	12.5%	
AV block	0	4	0.550
	0%	16.67%	
Medications			
Beta‐blocker	8	20	0.217
	100%	83.33%	
ARB or ACE inhibitor	8	21	0.294
	100%	87.5%	
Diuretic	8	20	0.217
	100%	83.33%	
Lipid‐lowering agent	5	12	0.539
	62.5%	50%	
Device			
CRT‐D	4	18	0.218
CRT‐P	4	6	

ARB, angiotensin receptor blocker; ACE, angiotensin‐converting enzyme; AV, atrioventricular; CMP, cardiomyopathy; COPD, chronic obstructive pulmonary disease; CRT‐D, cardiac resynchronization therapy defibrillator; CRT‐P, cardiac resynchronization therapy pacemaker; PISCO, pressure‐controlled intermittent coronary sinus occlusion; SD, standard deviation.

a Evaluated using the unpaired Student's *t*‐test.

b Fisher analysis was used if *n* < 5.

### Parameters during the intervention

In PICSO patients, the mean CSP before the intervention was 11.7 and 12.1 mmHg after the intervention. During the intervention, CSP systolic pressures rose 107% to mean 24.2 mmHg CSP with systolic peaks of 52 mmHg.

### Effects of pressure‐controlled intermittent coronary sinus occlusion on soluble factors secreted into cardiac veins

#### MicroRNA

**Figure 2:**
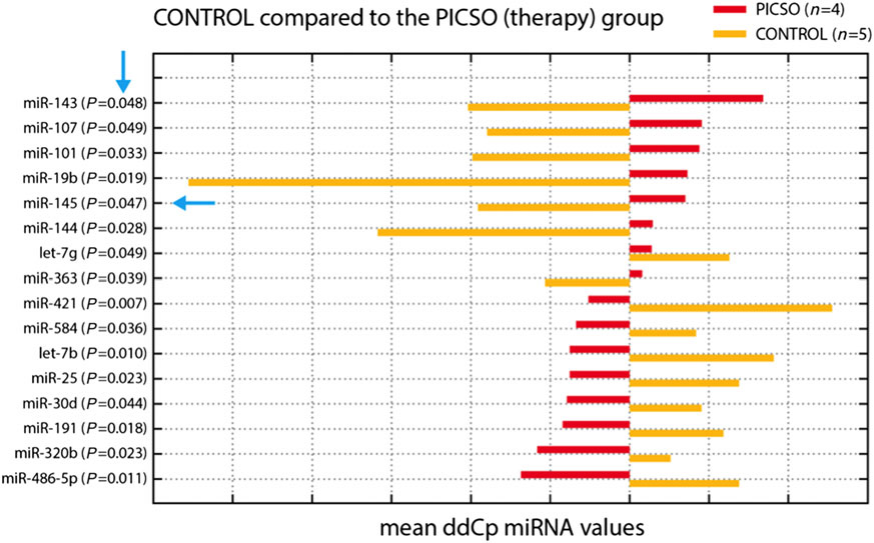
Differentially expressed microRNA (miRNA). Here, we show differentially expressed miRNA for both groups. Delta differences between first and second serum collection are shown as mean ddCP for both groups. After normalization of data to global mean, a delta Cq value is obtained (dCq). Differences in expression levels are calculated as dCq (1) − dCq (2) = ddCq. Only statistically significant patterns of miRNA expression are shown. miRNA 145 is involved in a double negative feedback loop with Krüppel‐like factor 4 inducing lineage restricted differentiation. PICSO, pressure‐controlled intermittent coronary sinus occlusion.

The time courses of microRNA in both groups were monitored revealing distinguished trends. This difference (as reported by the comparison of the difference normalized Ct post‐value minus normalized Ct baseline) was statistically significant for 16 of the tested microRNAs (*Figure* *2*). In the PICSO group, we observed a relative increase of miR‐19b, miR‐101, miR‐143, miR‐144, and miR‐145. A gene enrichment analysis of the data emphasized possible candidate pathways to belong to the Wnt pathway (miR‐101 and miR‐144), muscle organ development (miR‐101 and miR‐144) or muscle (miR‐143), aortic and coronary endothelial function (miR‐144), and muscle contraction (miR‐19b). In parallel, the concentrations of let‐7b, miR‐25, miR‐191, miR‐320b, miR‐421, and miR‐486‐5p were relatively reduced.

In the control group with resynchronization only, miR‐421, let‐7b, miR‐485‐5p, miR‐25, miR‐191, miR‐30d, miR‐584, and miR‐320b were increased, and miR‐19b, miR‐144, miR‐143, miR‐101, miR‐145, miR‐107, and miR‐363 were decreased.

Thus, these data suggest that PICSO treatment had several significant effects on the circulating plasma microRNA in comparison with the control group.

Clinical observation and the mechanotransduction effect induced by PICSO intervention indicate a domain change between epigenetic forces and genome, a transition of hemodynamic impulses into a pleiotropic molecular signalling pulse as suggested by significant changes in coronary sinus plasma levels of several microRNAs. Regulation of cardiogenic gene expression by microRNA has been shown to play a prominent role in heart development. Recent data have identified various microRNAs as critical regulators of the proper differentiation, proliferation, and survival of cardiomyocytes, endocardial cells, epicardial cells, and neural crest cells. The potential of influencing microRNA expression may therefore be utilized in management and prevention of cardiovascular diseases.^5^

A number of studies demonstrated that elevated levels of miR‐143 and miR‐145 play an important role in regulating several transcription factors like octamer‐binding transcription factor 4, sex*‐*determining region Y*‐*box 2, and Krüppel‐like factor 4 (KLF‐4) as well as promoting differentiation and simultaneously repressing proliferation of vascular smooth muscle cells. The significant increase in plasma levels of miR‐143 and miR‐145 in PICSO‐treated patients together with the significant up‐regulation of KLF‐4 is suggestive of activation of such pathways. There are also a series of other differentially up‐regulated microRNA influencing microRNA.

Whereas let‐7b, let‐7g, and miR‐30d have been found in some studies to be involved in epithelial mesenchymal transformation and angiogenesis as well as improvement of contractility and are therefore considered positive in regenerative molecular pathways; in our patient series, the levels are lower in PICSO than in controls, requiring careful interpretation in the context of multiple intertwined processes and ambivalent responses of individual evoked signals in context especially when induced in failing myocardium.

#### Transcription factors

**Figure 3:**
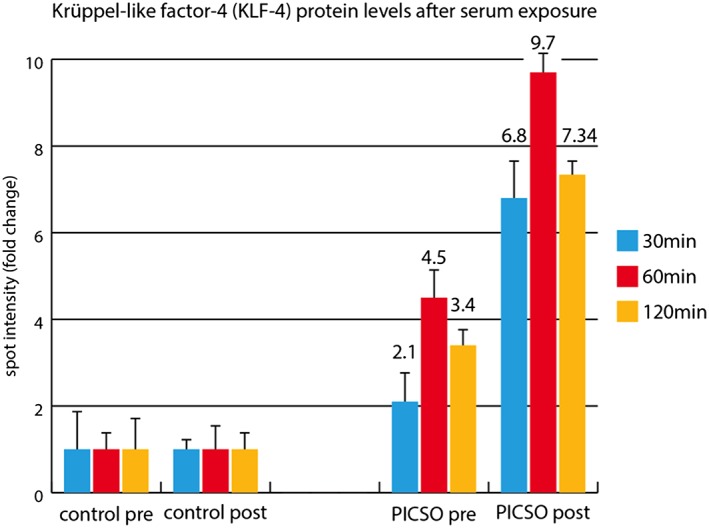
Krüppel‐like factor 4 (KLF‐4) in cardiomyocytes after serum exposure. Cardiomyocytes supravitally sampled from transplant recipient hearts were exposed to serum collected in three control and three pressure‐controlled intermittent coronary sinus occlusion (PICSO) patients according to protocol. Pre‐control and post‐control were set as baseline fluorescence intensity to compare signal differences in PICSO patients. Blue bar, 30 min; red bar, 60 min; orange, 120 min after treatment start. Groups are indicated on the bottom. Bars indicate fold change to control group as defined as 1. Mean fold change is given above error bars of standard deviation. Signalling of sera co‐cultured seem to peak at 60 min and decline thereafter.

Several transcription factors sampled from coronary venous blood reacted to the PICSO intervention and to lead testing (*Table* 2), suggesting group difference especially in 49 kDa MEF2C, a morphogen involved in cardiac morphogenesis, the development of myogenesis and vasculature, and increase in PICSO patients, whereas it remained unchanged in controls. As seen in spite of the small number, pre‐values and post‐values react in both groups to surgery and the intervention, showing a complex matrix, which deserves larger patient cohorts for meaningful interpretation. As reaction to soluble factors released, KLF‐4 was up‐regulated in explanted human myocardium from transplant recipients co‐cultured with post‐PICSO sera based on protein levels (*Figure* *3*).

**Table 2:** Transcription factors in cardiac venous blood

		Pre‐values		Post‐values			
Type	Group	Mean	SD	Mean	SD	*P*‐value	Change in %
39 kDa FGF5	PICSO	1590.06	767.70	1720.25	1195.02	0.3461	108.19
39 kDa FGF5	Control	5355.90	3002.89	5141.62	2637.02	0.2887	96.00
36 kDa FGF5	PICSO	2280.27	2214.61	2943.83	3000.44	0.2191	129.10
36 kDa FGF5	Control	3521.64	2600.49	2765.38	2449.93	0.1312	78.53
35 kDa FGF5	PICSO	4939.24	2099.17	5428.65	1682.89	0.1843	109.91
35 kDa FGF5	Control	4395.15	972.55	4987.40	1195.45	0.0858	113.48
33 kDa FGF5	PICSO	420.00	387.83	408.00	377.30	0.4518	97.14
33 kDa FGF5	Control	760.04	316.19	724.87	293.82	0.3898	95.37
60 kDa TBX5	PICSO	1765.94	863.50	1312.97	1055.07	0.0199	74.35 (*P* < 0.05)
60 kDa TBX5	Control	3923.35	2434.77	3420.49	2564.07	0.0392	87.18 (*P* < 0.05)
51 kDa TBX5	PICSO	15 583.03	9002.37	13 358.04	6304.52	0.1031	85.72
51 kDa TBX5	Control	18 691.01	3699.77	16 532.11	3648.23	0.0008	88.45 (*P* < 0.05)
41 kDa TBX5	PICSO	9052.13	7431.67	4229.24	4571.33	0.0343	46.72 (*P* < 0.05)
41 kDa TBX5	Control	8670.12	9768.61	4512.81	4529.47	0.0409	52.05 (*P* < 0.05)
48 kDa GATA4	PICSO	11 881.95	1853.15	9155.60	4549.00	0.2165	77.05
48 kDa GATA4	Control	20 211.56	8359.36	11 642.15	6247.43	0.0010	57.60 (*P* < 0.05)
52 kDa MEF2C	PICSO	2022.00	783.74	2424.36	1290.11	0.1877	119.90
52 kDa MEF2C	Control	2445.52	1038.12	2940.57	1197.48	0.0441	120.24 (*P* < 0.05)
49 kDa MEF2C	PICSO	8211.22	1830.25	10 197.42	1832.86	0.0232	124.19 (*P* < 0.05)
49 kDa MEF2C	Control	11 319.58	2698.46	11 750.39	3171.96	0.1896	103.81
39 kDa HAND2	PICSO	364.73	480.48	1041.79	555.15	0.1341	285.64
39 kDa HAND2	Control	936.69	664.02	1641.70	627.79	0.0336	175.27 (*P* < 0.05)
36 kDa HAND2	PICSO	1645.37	659.21	905.55	690.90	0.1317	55.04
36 kDa HAND2	Control	1285.81	800.58	796.52	442.24	0.0584	61.95
35 kDa HAND2	PICSO	2853.06	1721.25	2057.02	945.99	0.1313	72.10
35 kDa HAND2	Control	4247.16	864.58	3927.03	1265.71	0.1962	92.46
31 kDa HAND2	PICSO	11 503.93	3422.18	14 250.30	3086.66	0.1367	123.87
31 kDa HAND2	Control	9523.03	4450.82	13 858.94	4506.63	0.0032	145.53 (*P* < 0.05)

SD, standard diviation; PISCO, pressure‐controlled intermittent coronary sinus occlusion.

Transcription factors sampled from coronary venous blood in relation to pre‐interventional and post‐interventional values in both groups show a complex pattern of reactions in both groups. A potential interventional effect of PICSO can only be suspected in 49 kDa MEF2C. To allow sophisticated interpretation, a larger cohort of patients is needed.

### Proliferation results

**Figure 4:**
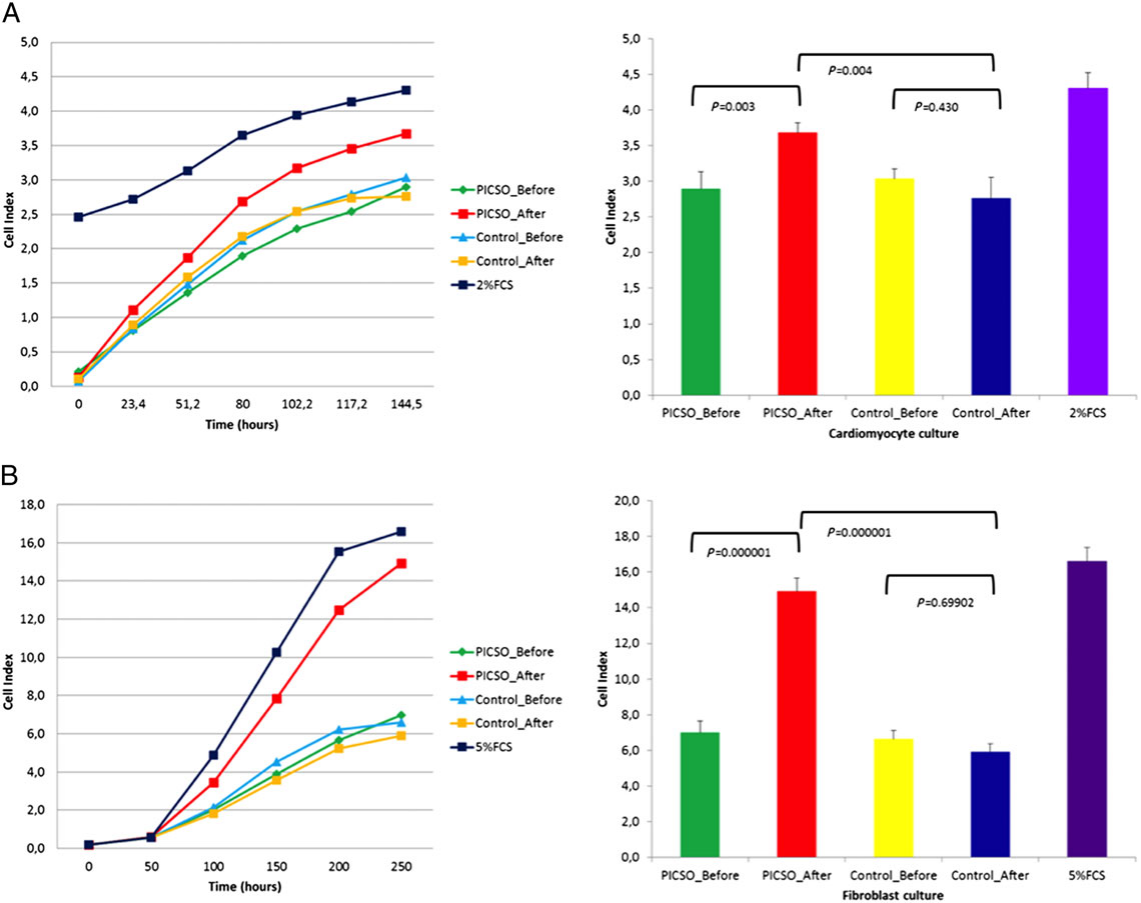
Real‐time cell index for (A) human primary cardiomyocytes sampled from an explanted heart from a patient with heart failure during transplantation and (B) commercially available fibroblasts cultured in the presence of PICSO (*n* = 5 patients) or non‐PICSO (*n* = 6 patients) sera from pre‐procedure and post‐procedure. Cells index was measured up to 250 and 144 h for fibroblast and primary cardiomyocytes, respectively. Student's *t*‐test significance is given in the graphs on the right for comparisons among groups at the end of the measurement periods. Note that post‐PICSO sera induced additional proliferation, whereas in post‐control sera, no change was evident.

There was a significant difference in the proliferation of human fibroblasts in the PICSO group compared with the control group. The growth curve generated via real‐time cell analysis RTCA software revealed that fibroblast growth before and after PICSO‐treated serum had distinctive trend for cell proliferation profile. Serum sample from after PICSO treatment showed significant increase in cell index immediately after sample addition. Results are depicted in (*Figure* *4*).

## Conclusions

The key element of our exploratory study is the association of a temporary pressure increase in coronary veins leading to molecular changes known to be involved in cardiac development. There appears to be a causative sequence between the haemodynamic, ‘epigenetic’ impulse of temporarily elevated coronary venous pressure via mechanotransduction and the activation of coronary venous vascular cells.^6^ As proposed in the ‘embryonic recall hypothesis,’ this activation and subsequent mechanotransduction via primary cilia and cytoskeleton of venous endothelium induce a burst of a differential pattern of microRNA in PICSO‐treated patients together with the induction of several transcription factors, as secreted morphogens may be the key phenomenon that connects the adult failing heart to innate but dormant cardiac developmental pathways.^7^ The up‐regulation of miR‐143 in PICSO‐treated patients, as well as the findings by Miyasaka *et al*. in the developing heart during the first heartbeat, supports our hypothesis in this context.^8^ In agreement to Marfella *et al*., investigating soluble factors as prognostic biomarkers in CRT patients, flow‐sensitive miRNA 145‐5p increased compared with controls in our verum group.^9^ Importantly, the effects reported relate to secretion of molecules, rather than *de novo* synthesis of morphogens, as the post‐procedure timing for assessment was limited to 20 min. However, long‐lasting signalling and the induction of a molecular cascade might lead via a domino effect to structural regeneration.^10^

In summary, PICSO modulated the expression of several relevant microRNA, which were detectable in blood of the coronary sinus. The resulting microRNA pattern indicates a situation favouring cardiac regeneration by action on cardiomyocytes and cardiac fibroblasts as well as by prevention of stress‐related antiangiogenetic effects.

Our observations also suggest that reconnecting adult failing hearts with cardiac development and therefore innate dormant process can be revived and eventually may be used to reverse the vicious cycle of severe symptoms in patients with otherwise untreatable heart failure.

We postulate that application of PICSO in a controlled trial in patients with severe chronic heart failure will substantiate the clinical significance of these molecular findings.

## Conflict of interest

W.M. is the inventor of PICSO and founder of Miracor. Miracor is presently exploring the clinical potential of PICSO in myocardial infarction. Miracor provided no funding for the ATOS I trial presented here. P.W.S. has served as consultant of Abbott, AstraZeneca, Biotronik, Cardialysis, GLG research, Medtronic, Sino Medical, Soc. Europa Dig. Publishing, Stentys, Svelte, Philips Volcano, St. Jude Medical, QualiMed, and Xeltis. No other co‐author had a conflict of interest.

## Funding

This work was supported by the Austrian Science Fund (P13274‐Med), Austrian Federal Ministry of Traffic, Innovation and Technology Vienna Austria (BMVIT GZ609.637/000‐III/12/2004), and the International Society of Coronary Sinus Interventions www.coronarysinus.com, Vienna Austria (educational grant 1/2009).
